# Proteomic Profiling of Plasma-Derived Biomarkers in Patients with Bladder Cancer: A Step towards Clinical Translation

**DOI:** 10.3390/life11121294

**Published:** 2021-11-25

**Authors:** Taoufik Nedjadi, Nada Albarakati, Hicham Benabdelkamel, Afshan Masood, Assim A. Alfadda, Jaudah Al-Maghrabi

**Affiliations:** 1 King Abdullah International Medical Research Center, King Saud Bin Abdulaziz University for Health Sciences, Jeddah 21423, Saudi Arabia; albarakatina@ngha.med.sa; 2 Proteomics Resource Unit, Obesity Research Center, College of Medicine, King Saud University, Riyadh 11461, Saudi Arabia; hbenabdelkamel@ksu.edu.sa (H.B.); afsmasood@ksu.edu.sa (A.M.); aalfadda@ksu.edu.sa (A.A.A.); 3 Department of Pathology, King Abdulaziz University, Jeddah 21589, Saudi Arabia; jalmaghrabi@hotmail.com

**Keywords:** bladder cancer, biomarkers, proteomics, plasma, 2D-DIGE, mass spectrometry

## Abstract

Background: Bladder cancer is a life-threatening disease and a major cause of cancer-associated complications. The main challenges confronted during the clinical management of bladder cancer are associated with recurrence and disease progression to the muscle-invasive phenotype. Improved early detection of the disease is of paramount importance to prevent disease progression and improve survival. Hence, novel clinically applicable biomarkers for early detection are warranted. Methods: In the current study, a comparative proteomic approach was undertaken using plasma samples to identify protein biomarkers associated with the muscle-invasive phenotype of bladder carcinoma. Isolated plasma proteins were depleted, DIGE-labeled, then subjected to conventional 2D electrophoresis followed by mass spectrometry for identification of differentially expressed proteins. Western blot was used for data validation. Results: Fourteen differentially expressed proteins with statistically significant changes in abundance between the cancer group and control group were identified. Three differentially expressed proteins were selected for validation, among which apolipoprotein A1 exhibited high specificity and sensitivity (AUC = 0.906). Ingenuity pathway analysis identified IFN-γ and TNF-α as the main signaling hub for the differentially regulated proteins. Conclusion: Our findings provide additional insight into understanding bladder cancer pathogenesis. Our data identified potential non-invasive plasma-derived biomarker proteins that merit additional investigation to validate its clinical usefulness to prevent bladder cancer progression.

## 1. Introduction

Bladder carcinoma (BC) is one of the most frequent malignancies with an estimated 450,000 new cases and more than 199,000 deaths in the year 2018, making it the tenth most common cancer worldwide [1]. At the first presentation to the clinic, 30% of the patients are diagnosed with muscle-invasive bladder cancer (MIBC) and metastatic cancer. The remaining two-thirds of bladder cancer patients are frequently diagnosed with non-muscle-invasive cancer (NMIBC) but have high rates (50–70%) of recurrence [2]. Importantly, a significant proportion of NMIBC patients (up to 30%) are prone to progression from the NMIBC to the MIBC phenotype [3]. The management of MIBC is very stringent and requires either surgical resection of the whole bladder or radical radiotherapy and provides a low five-year overall survival rate of <50% [4,5,6]. Hence, early detection of bladder cancer is of paramount importance for better disease management and improvement of patients’ survival. At present, there are no clinically applicable molecular tools for screening or early detection. Cytology and cystoscopy procedures, which are considered the gold standards for bladder cancer detection and surveillance, offer suboptimal sensitivity in addition to being invasive and expensive [7]. Moreover, the scoring system established by the European Organization for Research and Treatment of Cancer to predict the risks connected with bladder cancer progression and recurrence is currently insufficient and requires major improvement [8].

In the absence of a comprehensive approach for detection, progression monitoring and risk stratification of MIBC patients, thorough investigations have been conducted to develop reliable biomarkers [9,10]. Thus far, several FDA-approved biological tests, mostly protein-based assays and a DNA-based assay using body fluid, have been developed for diagnostics and follow-up of bladder cancer. However, none of these markers have been accepted in clinical practice due to limited sensitivity and specificity [11]. This situation necessitates the search for alternative comprehensive biological tools to develop a reliable non-invasive biomarker for early detection of bladder cancer and better understanding of the underlying molecular mechanisms associated with its progression.

**Figure 1:**
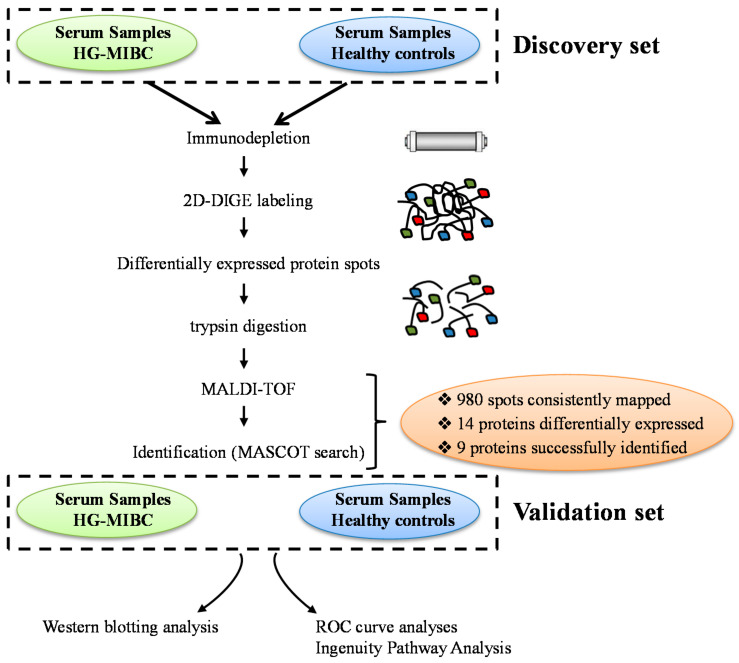
An overview of the workflow for plasma protein analysis. The flowchart illustrates the different steps involved the analysis, the patient populations, and the methods used in the discovery and the validation cohorts.

Plasma-derived protein biomarkers are particularly attractive for being relatively painless and easy to collect [12,13]. Additionally, it is well-perceived that changes in the protein profile significantly reflect the physiological changes and the pathological alterations occurring during the carcinogenic process [14,15], which then can be used as a diagnostic or prognostic tool in clinical practice [16,17,18,19]. In this context, serum levels of the S100A8 and S100A9 proteins were reported as promising prognosticators in bladder cancer patients [20]. Similarly, several glycans node markers were identified as prognostic indicators in the plasma of MIBC patients [21]. In the current study, patients samples (discovery and validation) were utilized to identify novel candidate marker proteins which are associated with MIBC progression. A proteomics-based approach, 2D-difference gel electrophoresis (2D-DIGE) followed by matrix-assisted laser desorption/ionization-time of flight (MALDI-TOF) mass spectrometry was adopted for biomarker detection. Our strategy uncovered several promising biomarker candidates for bladder cancer which were further validated using Western blotting analysis in an independent cohort of plasma samples from high-grade MIBC patients and healthy donors. Ingenuity pathway analyses were performed to unravel functionality and molecular pathways of the identified proteins. The detailed study design is illustrated in Figure 1.

## 2. Materials and Methods

### 2.1. Patient and Samples Collection

Study participants included newly diagnosed bladder cancer patients from King Faisal specialist hospital and Research Center (KFSH&RC, Jeddah, Saudi Arabia) and King Abdulaziz University Hospital (KAUH, Jeddah, Saudi Arabia). The inclusion criteria included all patients with high-grade (HG) muscle-invasive tumors able to sign informed consent. All samples were collected during routine procedures. The collection and analysis of all samples were ethically approved by the institutional Review Board Committee at KFSH&RC (reference No: RC-J/36/36) and by the Institutional Research Ethics Committee at KAUH (reference No. 149-04).

A total of 51 patients (cancer and controls) were recruited in the study. Informed consent was obtained from bladder cancer patients (age range 51–91 years, mean age 69 years) and healthy controls (age range 43–60, mean age 51 years). The blood samples were drawn from MIBC patients prior to any therapeutic intervention and chemo-radio therapy. EDTA-containing tubes were used to collect blood samples. Plasma was prepared by centrifugation at 2500× *g* for 20 min at 4 °C. Afterwards, the plasma samples were aliquoted in new cryotubes and promptly stored at −80 °C until further use.

### 2.2. Depletion of Abundant Proteins

Prior to any proteomics experiments, plasma samples were carefully processed to deplete highly abundant proteins including immunoglobulins, albumin, alpha-1 antitrypsin and transferrin that may interfere with MS analysis and biomarker detection. Depletion was performed using a multiple affinity removal system, Top-20 Depletion ProteoPrep spin columns (Sigma), according to the manufacturer’s instructions and protocol. An example of whole undepleted plasma and depleted fraction is shown in Supplementary Figure S1.

### 2.3. Two-D DIGE Labeling

After the depletion step, a TCA/acetone precipitation was performed to remove interfering compounds and minimize plasma protein degradation. Labeling of proteins with cyanide dyes was done as described previously. Typically, depleted plasma proteins were mixed with ice-cold acetone/TCA (10% w/v) in a ratio of 1:4 and vortexed for 15 s. Protein precipitation was achieved after overnight incubation at −20 °C. The mixture was then centrifuged at 2000× *g* for 15 min at 4 °C. The resulting pellet was solubilized in a labeling buffer containing 30 mM Tris–HCl (pH 8.5), 7 M urea, 4% CHAPS, 2 M thiourea. Protein concentrations were determined in triplicate using a 2D-Quant kit (GE Healthcare, Chicago, USA), and 50 µg from each sample was used for the labeling step. The proteins from healthy control, HG cancer, or internal standard were labeled with 400 pmol of either Cy3, Cy5, or Cy2 dyes, respectively, and loaded on the gels (Table S1).

### 2.4. Two-Dimensional Gel Electrophoresis and Mass Spectrometry Protein Identification

First, dimensional separation was achieved through rehydration of the immobilized pH gradient from pH 3–11 (IPG) strips using individually labeled proteins. Isoelectric focusing (first dimension) was performed using a Multiphor II apparatus. The second dimension was established by proteins separated on 12.5% (SDS-PAGE) gels using an Ettan Dalt Six device. The three 2D gels were scanned using appropriate wavelengths and filters specific for Cy2, Cy3, and Cy5 dyes. Images were captured, and differentially expressed proteins were analyzed using Progenesis Same Spots v.3.3 software (Nonlinear Dynamics Ltd., Newcastle, UK). Differences were also checked manually before applying the statistical criteria (ANOVA test, *p* ≤ 0.05 and fold ≥1.5). At this stage, filtration and normalization of spot volumes/protein abundance was calculated for statistical analysis. Protein spots that showed significant difference in expression were submitted for mass spectrometry identification.

Coomassie-stained protein spots were excised, destained, and subjected to overnight trypsin digestion at 37 °C. A MALDI target (384 MTP Anchorchip; 800 m Anchorchip; Bruker Daltonics, Bremen, Germany) was spotted with a mixture of tryptic peptides (1 uL) derived from each protein. MALDI-TOF (MS) spectra were obtained using an UltraflexTerm TOF mass spectrometer equipped with a LIFT-MS/MS device (Bruker Daltonics, Bremen, Germany) at reflector and detector voltages of 21 and 17 kV, respectively. Using Flex Analysis software, the PMFs were assessed (version 2.4, Bruker Daltonics). BioTools v3.2 was used to interpret MS data (Bruker Daltonics). The Mascot search algorithm (v2.0.04, updated on 09/05/2020; Matrix Science Ltd., London, UK) was used to search the peptide masses. Mascot parameters were as follows: fixed cysteine modification with propionamide, variable modification due to methionine oxidation, one missed cleavage site (i.e., in the case of incomplete trypsin hydrolysis), and a mass tolerance of 100 ppm. Identified proteins were accepted as correct if they showed a Mascot score greater than 56 and *p* < 0.05.

### 2.5. Protein Interaction and Network Analysis

Only proteins showing significant change in the expression pattern were imported into Ingenuity Pathway analysis (IPA) software (Ingenuity^®^ Systems, http://www.ingenuity.com, accessed on 10 August 2021) and were subjected to regulatory network analysis and functional annotation. All information related to biological process, subcellular localization, protein interactions, pathways, and networks involving the bladder cancer-associated proteins were determined.

### 2.6. Data Validation: Western Blot Analysis

Samples from an independent cohort of HG cancer patients and healthy controls was used for data validation. Plasma samples were diluted 1:4, and 2 µL of plasma was loaded and separated on 8% SDS-polyacrylamide gels. Proteins were then transferred into nitrocellulose membranes and blocked with 5% non-fat milk, 1% (*v*/*v*) Tween-20 in TBS for 1 h at room temperature. The membranes were incubated with the appropriately diluted primary antibodies (gelsolin, apolipoprotein A1, and inversin) overnight at 4 °C. HRP-conjugated secondary antibodies were used for detection. The bands were visualized with an enhanced chemiluminescence reagent. Equal sample loading was determined by separating plasma samples on 8% PAGE gels and stained with Coomassie blue stain.

### 2.7. Statistical Analysis

All statistical analyses were carried out using Fisher’s exact test. The densitometry data are shown in the graph with a mean ± standard deviation (SD). A *p*-value less than 0.05 was considered statistically significant. Receiver operating characteristics (ROC) were used to measure the performance of the target proteins and to assess the specificities and sensitivities. The diagnostic value of the candidate biomarkers was assessed by calculating the respective areas under the curve (AUC). The data was analyzed and graphs were generated using SPSS software (SPSS, V.25, Armonk, NY, USA).

## 3. Results

### 3.1. Patients’ Samples

In order to analyze the protein signature associated with bladder cancer progression, plasma samples were divided into two cohorts: discovery cohort (N = 8) and validation cohort (N = 43). The discovery cohort was grouped into two groups: the first one harboring patients with high-grade muscle-invasive tumors (four samples) and the second group for healthy donors (four samples). Additional characteristics of both cohorts are presented in Table 1. Plasma samples were subjected to immunodepletion to reduce the amount of highly abundant proteins (IgG and albumin) that might interfere with mass spectrometry analysis (Supplementary Figure S1). This step was completed using an antibody-based affinity column to reduce the complexity of the samples and to increase the chance to identify low-abundant proteins. The DIGE experiment was performed to delineate the differential protein expression pattern. Protein fractions (50 µg) were then labeled as follows: healthy control group with Cy3 dye and HG group with Cy5 dye. The Cy2 dye-labeled sample was run in parallel with the other gels to remove gel-to-gel variation. This sample, which consists of pool mixture of control and HG samples, is the internal standard of the experiment.

### 3.2. Proteomic Analysis and Identification of Candidate Biomarkers

**Figure 2:**
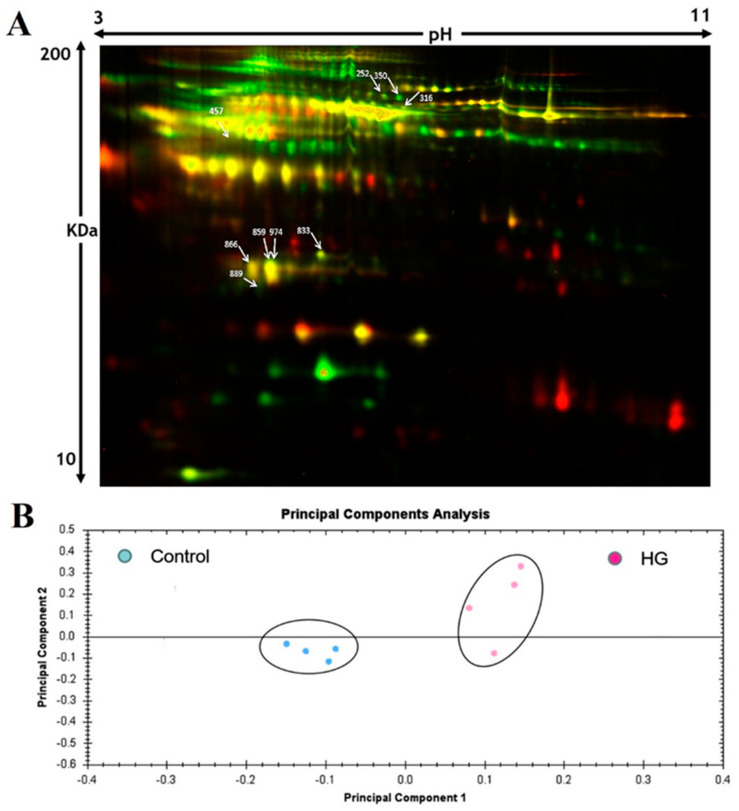
A representative overlap 2D-DIGE image. (**A**) The proteome maps obtained from two-dimensional proteome map analysis of plasma samples from bladder cancer patient and healthy controls. Vertically, MW range 10–200 KDa and horizontally, pH range 3–11. The differentially expressed spots are denoted by arrows. (**B**) Principal component analysis (PCA) of the abundance of differentially expressed proteins. Colored dots represent the analyzed gels from high-grade cancer and controls.

The changes in global protein expression profiles between high-grade muscle-invasive cancers and healthy controls in the discovery cohort were analyzed using 2D-difference gel electrophoresis (2D-DIGE). Four different gel images corresponding to the HG and healthy control groups as well as the internal standard were generated for each gel (Figure S2). Twelve obtained images were analyzed using Progenesis Same Spot software to mark differentially expressed proteins. A total number of 980 spots were reliably mapped between the two groups. A representative 2D-DIGE gel is shown in Figure 2A. Changes in spot intensities were analyzed using the same software, and 14 protein spots with a significant statistical change in abundance (ANOVA test *p* < 0.05; fold change > 1.5) that were consistently disregulated between the high-grade and control group were aligned for further analysis. Differentially expressed protein spots were excised and digested for identification by mass spectrometry. Using MALDI-TOF spectrometry, nine spots were successfully identified and matched using the MASCOT peptide mass fingerprints (PMF) to entries in the SWISS-PROT database with high confidence. Sometimes, variants of the same protein were detected at several locations on the gel. Our data revealed that of the nine proteins, eight were downregulated and one upregulated in patients with high-grade bladder cancer compared to the control group. The identification profile characteristics of the differentially expressed protein spots are given in Table 2.

### 3.3. Pattern of Variation of Differentially Expressed Proteins

Extended data analysis was carried out using principal component analysis (PCA) on Progenesis SameSpot software to evaluate the trend of differentially expressed proteins and their behavior in HG cancer patients and healthy controls. All eight gels images were grouped into two groups according to whether they are HG cancer or not, and multivariate analyses of abundant proteins that were present on all gel images and identified by MS were selected for such analysis. PCA plot of the two first principal components revealed that the two groups (HG and control) clustered distinctly from each other, and the selected spots exhibited 56.4% abundance variability based on the presence of the disease and indicating differences in the nature of plasma samples between cancer and control (Figure 2B).

### 3.4. Functional Characteristics and Pathway Analysis

**Figure 3:**
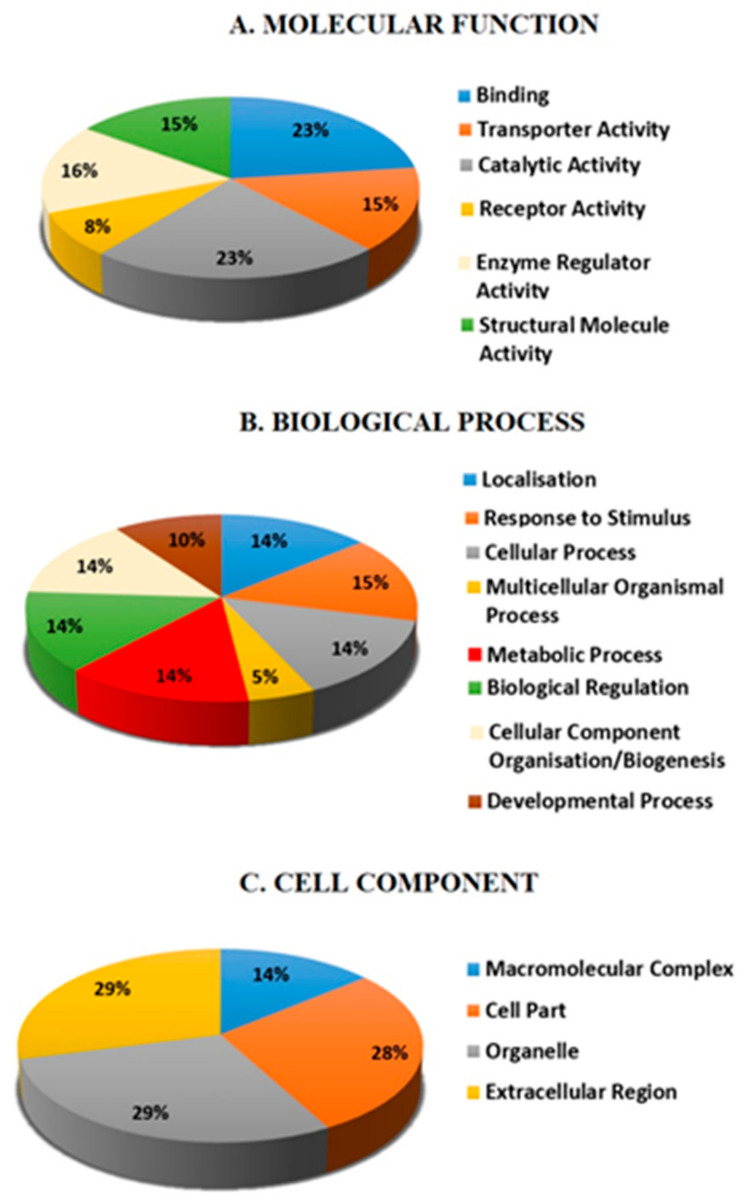

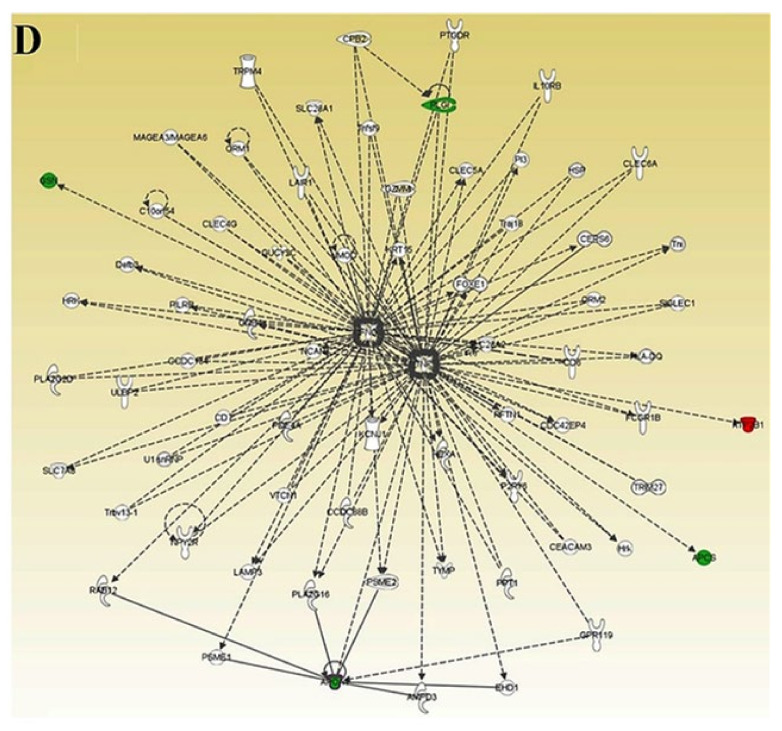
Protein interaction network and function using Ingenuity Pathway Analysis (IPA). Representative pie charts indicating the molecular and biological functions (**A**,**B**) and subcellular localization (**C**) of identified proteins. (**D**) The major protein interaction network of identified proteins. Direct interactions are represented as solid lines, whereas indirect interactions appear as dotted lines.

Ingenuity Pathways Analysis (IPA) was performed to understand the functional significance of the differentially expressed proteins. IPA is a powerful tool commonly used to determine protein–protein interactions and accurately predict signal transduction pathways. PANTHER (protein analysis through evolutionary relationships) classification system (http://www.pantherdb.org, accessed on 10 August 2021) was used for classification of proteins after MS according to their function (Figure 3A–C). The predominant functions of the identified proteins involved binding activity (23%), catalytic activity (23%), enzyme regulator activity (16%), structural activity (15%), and receptor activity (8%). The main biological processes include response to stimuli (15%), cellular process (14%), metabolic process (14%), and development process (10%). Most of the proteins are a component of different cellular compartments, including the organelles (29%), the extra-cellular region (29%), a cell part (28%), and a macromolecular complex (14%).

Pathway and network analysis of differentially expressed proteins in HG bladder cancer using Ingenuity Pathway Analysis revealed numerous common proteins linked together, with IFN-γ and TNF as important hubs (Figure 3D). Both cytokines (IFN-γ and TNF) play pivotal regulatory roles in cellular homeostasis, immune response, and cancer progression. Our pathway map exhibits the relationship between Apo A1 and TNF, which acted as the central mediator in this network, and the interconnection between gelsolin and IFN-g (Figure 3D). Hence, both proteins (Apo A1 and gelsolin) were selected for further validation. In addition, inversin was also selected for further validation, as this is the first study to report an elevated inversin level in bladder cancer and could serve as novel biomarker for bladder cancer progression.

### 3.5. Data Validation

**Figure 4:**
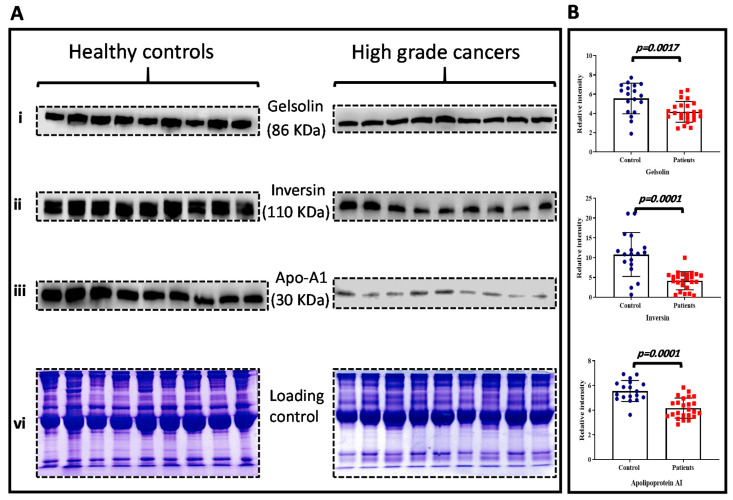
Biomarker validation using immunoblotting analysis. The expressions of selected differentially expressed proteins were validated in an independent cohort. Plasma samples were incubated with antibodies against (**Ai**) gelsolin, (**Aii**) inversin, and (**Aiii**) apolipoprotein A1. Equal protein loading was verified by staining the gels with Coomassie Brilliant Blue (**Avi**). The relative quantification of the expression level was performed by densitometry analysis of each protein normalized to β-actin (**B**). Error bars represent standard error of the mean. n = three replicates of three independent experiments for each group.

Proteins with a significant number of changes in cancer compared to those in healthy controls were considered as proteins of interest that might act as potential biomarker candidates. Data validation was carried out using immunoblotting analysis on plasma samples from independent cohorts of high-grade muscle-invasive bladder patients (n = 25) and healthy controls (n = 18). Three candidate proteins (inversin, gelsolin, and apolipoprotein A1) were selected for the validation study, all of which were downregulated in HG patients compared to healthy controls. These proteins were selected because they exhibited the largest difference in expression between HG cancer compared to healthy controls, and they were involved in other cancers development. The results collected from Western blotting analysis for all three proteins was consistent with the previous data and confirm the behavior observed in 2D-DIGE analysis. A representative immunoblot for each tested protein is presented in Figure 4A. For quantitative purpose, Coomassie blue staining was performed to ensure equal protein loading (Figure 4A). Densitometric analysis was undertaken for each of the specific immunoreactive band using Image J software, and the resulting mean values ± SD comparing HG cancer vs. control are presented in Figure 4B. Interestingly, all three proteins were significantly downregulated in the HG cancer group. This finding confirmed the 2D-DIGE results.

### 3.6. Diagnostic Efficiency of the Plasma Biomarkers

**Figure 5:**
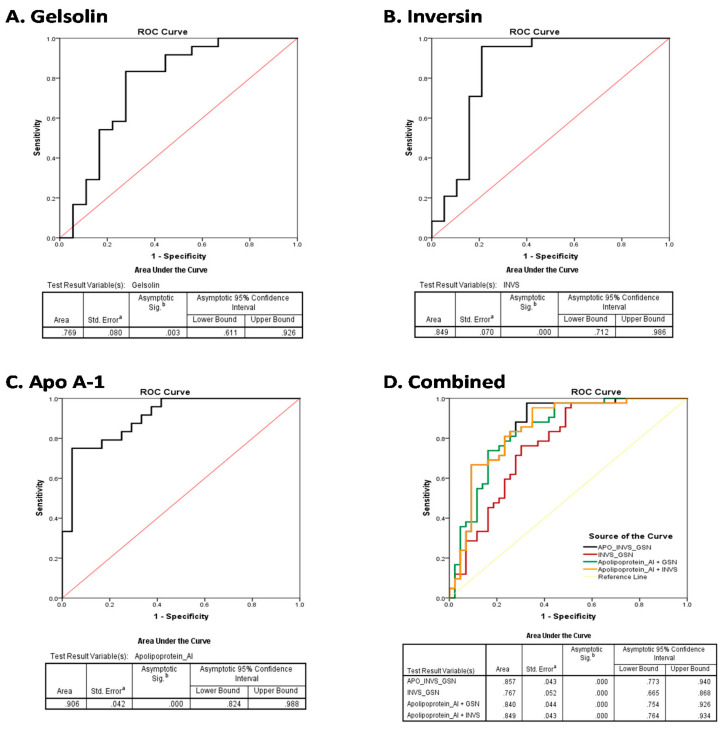
Diagnostic performance of the candidate biomarkers. Receiver operating characteristics (ROC) curves show gelsolin (**A**), inversin (**B**), and apolipoprotein A1 (**C**), independently or in combination (**D**) in the validation dataset. The respective areas under the curve (AUC) are shown by the estimate with 95% confidence interval. ^a^ Under the nonparametric assumption; ^b^ Null hypothesis; True area = 0.5.

The diagnostic performance of the three candidate proteins was assessed by sensitivity, specificity, and area under the curve (AUC). The ROC curves of the protein are shown in Figure 5. The concentration levels of the three circulating proteins inversin, gelsolin, and apolipoprotein A1 identified high-grade bladder cancer with an AUC > 0.84 (95% confidence interval 71–98), 0.76 (95% confidence interval 61–92%), and 0.90 (95% confidence interval 82–98), respectively. The combined panel of all three proteins did not show any significant improvement in accuracy over the individual proteins (Figure 5).

## 4. Discussion

Despite the impressive development in cancer genomics and therapy, our knowledge pertaining to urothelial bladder carcinoma biomarkers discovery is still lagging behind. Nowadays, cancer proteomics is widely used and is expected to enhance our understanding of the molecular mechanisms associated with cancer development and progression [22]. It offers advantages to identifying potential biomarker candidates for early detection, prognostication, predicting responses to treatments, and patients risk stratification. This is especially important for advanced forms of bladder cancer, where the curative procedure for muscle-invasive bladder cancer (MIBC) patients involves radical cystectomy with a five-year survival rate of <50% [23]. Therefore, identifying novel biomarkers is critical to improving diagnosis, prognosis, and treatment of MIBC. In our investigation, we adopted a proteomic approach to identify plasma protein biomarkers associated with high-grade tumors from advanced-stage bladder cancer patients (MIBC). Two-dimensional difference gel electrophoresis (2D-DIGE), which uses up to three fluorescent tags for protein labeling and is a useful tool to ascertain cancer biomarkers [24], followed by MALDI-TOF mass spectrometry for protein identification were performed. The technique is highly sensitive and allows the simultaneous identification and quantification of differentially expressed proteins between disease and control samples [25].

In this study, the proteomic profile of plasma specimens from high-grade muscle-invasive cancer-affected patients was examined and compared with that from healthy volunteers. Approximately 980 protein spots were successfully matched between the two groups on each gel, and out of 14 differentially expressed proteins, the identity of nine was unveiled using MALDI-TOF mass spectrometry (Table 2). Of the nine proteins, three were selected for further validation using Western blot analysis because they exhibited significant *p*-values of decrease in cancer compared to healthy controls. The identification of the same proteins at different positions may correspond to their isoforms or their post-translational modifications as a result of acetylation, methylation, phosphorylation, or glycosylation that can either shift the protein right or left, depending on the isoelectric point (PI), or up and down depending on the modifications in molecular weight (MW). Our previous work has also reported the identification of such protein isoforms differing both in size and isoelectric point in plasma proteomics [26,27]. The selected proteins (gelsolin, inversin, and Apo A1) showed a higher protein score and sequence coverage compared with those of the other identified proteins. Moreover, previous reports revealed that changes in serum levels of all three proteins have been remarkably altered in various solid tumors which support our findings. Additionally, gelsolin, inversin, and Apo A1 have been associated with a poor outcome [28,29,30].

Gelsolin is a ubiquitous protein expressed in the cytoplasm of most mammalian cells and in the extracellular fluids such as plasma. It is an 82 KDa calcium-binding actin-capping protein with multifunctional regulatory roles of cellular structure and metabolism [31]. It is well-perceived that malignant cells are characterized by abnormal arrangement of the actin cytoskeleton [32]. As an actin filament-severing and capping protein, it was reported that gelsolin is directly involved in cancer development and progression [33]. It is considered as a potential biomarker for inflammatory conditions and several disorders, including cancer. Downregulation of gelsolin protein expression in most malignancies is attributes to gene inactivation mediated by epigenetic modifications or impairment of promoter activity regulated by activating transcription factors or by ubiquitin-proteasomal degradation, as reported in pancreatic cancer [34,35,36]. In agreement with our data, a recent article by Chiu et al. (2020) revealed that a reduced circulating level of plasma gelsolin could be an independent diagnostic biomarker with sensitivity (82.7%) and specificity (95.6%) in head and neck cancer. The authors conclude that gelsolin may serve as an independent predictor of clinical outcomes and a novel biomarker for the early detection of head and neck cancer [37] and in bladder cancer [38]. Although in our study gelsolin exhibited low potency to discriminate between MIBC and the control group (AUC 0.76) among the three biomarkers, it still holds great promise to provide clinically applicable information. Interestingly, a reduced plasma gelsolin level was also recognized as a potential biomarker in precancerous conditions such as inflammation, sepsis, and HIV through releasing of inflammatory mediators, which suggests the clinical usefulness of recombinant gelsolin [39,40]. Furthermore, a reduced serum level of gelsolin is well-documented in ovarian cancer. In combination with lumican, gelsolin displays high accuracy in distinguishing pancreatic cancer from chronic pancreatitis (95% specificity) [41]. Downregulation of gelsolin is reported in breast cancer patients harboring a BRAC1 mutation and those receiving doxorubicin chemotherapy [42]. The broad spectrum of clinical conditions in which gelsolin is associated suggests that it is a universal predictor for general health [39].

Another important aspect of the current study is the identification of inversin and a potential marker associated with MIBC. Inversin, also known as nephrocystin-2, is a 110 KDa ciliary protein encoded by the INVS gene. The protein is required for normal cell cycle progression and tissue homeostasis and critically regulates renal developmental processes and establishment of the left-right axis in vertebrates [43,44]. Mutations of the INVS gene have been linked with polycystic kidney disease (nephronophthisis II), which is an autosomal recessive disorder, through activation of the sonic hedgehog pathway and/or negative regulation of the Wnt pathway [45,46]. The involvement and the biological role of inversin in human malignancies remains largely underinvestigated. Thus far, one article reported the association between overexpression of cytoplasmic inversin and disease aggressiveness and a poor outcome in non-small lung cancer by upregulating MMP2/MMP9 and activating the non-canonical Wnt (PCP/JNK) signaling pathway. The exact mechanism by which inversin could increase cancer invasion is at its infancy, however, modulating the epithelial–mesenchymal transition (EMT) by impairing E-cadherin expression and upregulating N-cadherin and Vimentin is a possible mechanism [29]. Pisamai and co-authors proposed a link between inversin and chemotherapy (cisplatin or doxorubicin) via proteins named calmodulin (CALM2), TP52, or nitric oxide synthase 3 (NOS3) in oral melanoma [47]. In bladder cancer, the expressions and biological functions in tumors have not been explored. The intracellular localization of inversin to the primary cilia of renal epithelial cells has been intensively studied [48,49], however, the soluble form of the protein has never been investigated. To our knowledge, this is the first study that investigates the expression of inversin in plasma samples in bladder cancer. The significant downregulation of inversin speculates that it may serve as a potential candidate biomarker for muscle-invasive bladder cancer. Further analysis is required to clarify the biological significance of inversin in bladder cancer. Furthermore, ROC curves were generated to evaluate the diagnostic value of inversin in MIBC. Analysis of the AUC (0.849) indicated that inversin could serve as a favorable diagnostic biomarker in high-grade muscle-invasive tumors.

Apolipoprotein A1 (Apo A1) is a major multifunctional regulatory protein component of high-density lipoproteins (HDL). Various studies have highlighted a wide range of biological properties of Apo A1, including inflammation, immunity, cancer, and metabolic disorders [50]. The role of Apo A1 in carcinogenesis has been studied extensively with particular emphasis on its anti-tumor properties in mouse models of cancers. Increased expression of Apo A1 impairs tumor growth and improves survival rate in transgenic mice [51,52]. High serum Apo A1/HDL levels have been associated with a decreased risk of several cancers and long survival in patients with solid tumors [53,54]. An inverse relationship between the plasma levels of the Apo A1 protein and tumor development has been reported in patients with gastric cancer, pancreatic cancer, and ovarian cancer [55]. In bladder cancer, the relationships between serum Apo A1 level and the disease remains unclear. Li et al. (2011) reported very high levels of Apo A1 in urine samples of patients with bladder cancer, particularly in those diagnosed with an aggressive phenotype, suggesting its usefulness as a potential non-invasive diagnostic and screening biomarker in bladder cancer patients [56,57]. The blood levels of Apo A1 have never been investigated in the context of bladder cancer. In consistence with previous data, we found that the plasma expression of Apo A1 was significantly downregulated in MIBC patients compared with that in the control group. Several studies reported that a reduced serum Apo A1 level correlates with disease progression, metastasis, and recurrence in many cancer types [55]. Furthermore, a plethora of studies have proven the anti-inflammatory features of Apo A1 by modulating the innate immune system [58]. Our study shows that Apo A1 has good discrimination ability between MIBC and controls (AUC 0.906). Combining the markers into different panels did not improve the discrimination provided by Apo A1 alone. However, careful interpretation of this data has to be taken given that Apo A1 is ubiquitously expressed with possible interference with other confounding factors. Taken together, our findings support the possible involvement of Apo A1 in bladder cancer progression given that MIBC represents an advanced stage of bladder cancer and is highly immunogenic with a poor outcome. Likewise, the plasma Apo A1 level could be used as a non-invasive diagnostic and screening biomarker for advanced bladder cases. Indeed, the exact mechanism of apolipoprotein A1 function in bladder carcinogenesis is yet to be clarified.

## 5. Conclusions

Our study showed that 2D-DIGE coupled with mass spectrometry is a powerful technique for the identification of biomarker proteins related to aggressive forms of urothelial bladder cancer. Our data identified nine plasma proteins, including serum amyloid P-component, mesoderm development candidate-1, plasma membrane calcium-transporting ATPase-1, plasminogen, gelsolin, inversin, and apolipoprotein A1. The last three proteins could serve as potential biomarker candidates for bladder cancer progression, providing novel insight into the diagnosis of bladder cancer. However, the relatively small sample size of our cohort as well as scarcity of clinicopathology are the main limitations of our study. Additionally, the lack of age-matching between cancer and healthy control groups that restricts the conduction of a comprehensive correlation and consistent comparison between groups is a drawback of the current investigation. All limitations need to be carefully considered to draw meaningful conclusions. Despite its limitations, our study has a major strength of using a rational approach that complements other studies to identify novel non-invasive biomarker proteins. The current data will eventually encourage subsequent prospective large-scale multicenter studies to validate our findings. Functional validation of the targets will further substantiate their involvement in the pathophysiology of bladder cancer progression and represents a potential therapeutic target to curtail the aggressive phenotype of bladder cancer.

## Figures and Tables

**Table 1:** Clinical and pathological characteristics of the study population.

Characteristics		MIBC Patients	Healthy Controls
**Number of subjects**		29	22
**Age**	(median/range)	67/(51–91 y)	51/(43–60 y)
**Weight**	(median/range)	67/(51–94 y)	74/(59–88 y)
**Gender**	Male	92.0%	83.3%
	Female	8.0%	16.7%
**Stage**	pT1	0%	N/A
	pT2	96.5%	
	pT3	0%	
	pT4	3.5%	
**Tumor grade**	High grade	100.0%	N/A
**Metastasis**	Lymph node	3.5%	N/A
**Vascular Invasion**	No	70.0%	N/A
	Yes	30.0%	
**Status**	Alive	100.0%	N/A
	Dead	0.0%	
**Recurrence**	No	33.3%	N/A
	Yes	66.7%	
**Smoking**	No	100.0%	N/A

**Table 2:** Identified proteins with changes in abundance between bladder cancer and control samples. The table shows the average ratio values for control and cancer with their corresponding levels of fold changes and one-way ANOVA (*p*-value < 0.05). ^a^ Theoretical isoelectric point; ^b^ theoretical relative mass; ^c^ coverage (%), ^d^ MASCOT score.

Spot No	Accession No	Protein Name	MASCOT ID	Pi ^a^	MW ^b^	Cov% ^c^	Score ^d^	*p*-Value (ANOVA)	Fold Change	Expression HG/Control
**350**	Q9Y283	Inversin	INVS	9.43	118837	61	58	0.002	1.5	Down
**833**	P02743	Serum amyloid P-component	SAMP	6.10	25485	30	60	0.028	2	Down
**889**	Q9H1K6	Mesoderm development candidate 1	MESD1	8.53	38533	34	58	0.030	2.8	Down
**859**	P02647	Apolipoprotein A-I	APOA1	5.56	30759	49	129	0.033	1.9	Down
**866**	P02647	Apolipoprotein A-I	APOA1	5.56	30759	56	145	0.044	1.7	Down
**457**	P20020	Plasma membrane calcium-transporting ATPase 1	AT2B1	5.73	139637	19	66	0.030	1.7	Up
**316**	P06396	Gelsolin	GELS	5.90	86043	51	62	0.052	1.5	Down
**252**	P00747	Plasminogen	PLMN	7.04	93247	40	149	0.0259	1.6	Down
**974**	P02647	Apolipoprotein A-I	APOA1	5.56	30759	59	160	0.038	1.8	Down

## Data Availability

The datasets used and/or analyzed during the current study are available from the corresponding author on reasonable request.

## Supplementary Materials

The following are available online at https://www.mdpi.com/article/10.3390/life11121294/s1; Figure S1: Depletion of plasma samples. Eight percent Coomassie blue-stained SDS gel showing 6 µg of whole plasma and depleted fractions from high-grade muscle-invasive bladder cancer patients (HG-MIBC) and healthy controls (HC); Figure S2: Protein expression profiling by 2D-DIGE: Images A and B for high-grade tumor and healthy control labeled with Cy3 and Cy5 dyes, respectively. Pool samples (internal standard) was labeled with Cy2 dye (C). Image D is an overlay of 2D-DIGE gels, Figure S3: Determining the sample size number. Pilot study of the power analysis using a group of samples (four replicates). A sample size of four was determined as sufficient to give a power of 80% by software Progenesis SameSpots Non Linear dynamics, Table S1. Experimental design for Cyanine Dye labeling of depleted plasma proteins for DIGE analysis. Discovery set, four high-grade cancer (HG/MIBC) and four control samples run on four 2D-PAGE gels, internal standards were used for normalization and created by pooling an equal amount of samples.
