# Supplementary material for: Proteomic Profiling of Plasma-Derived Biomarkers in Patients with Bladder Cancer: A Step towards Clinical Translation
Source: Life (Basel). 2021 Nov 25;11(12):1294. doi: 10.3390/life11121294 (PMC8704559; doi:10.3390/life11121294)
Supplement: Supplementary file 1 [file life-11-01294-s001.zip › life-1402174-supplementary.pdf]

life

Supplementary Material: Proteomic Profiling of Plasma-derived Biomarkers in Patients with Bladder Cancer: A Step towards Clinical Translation

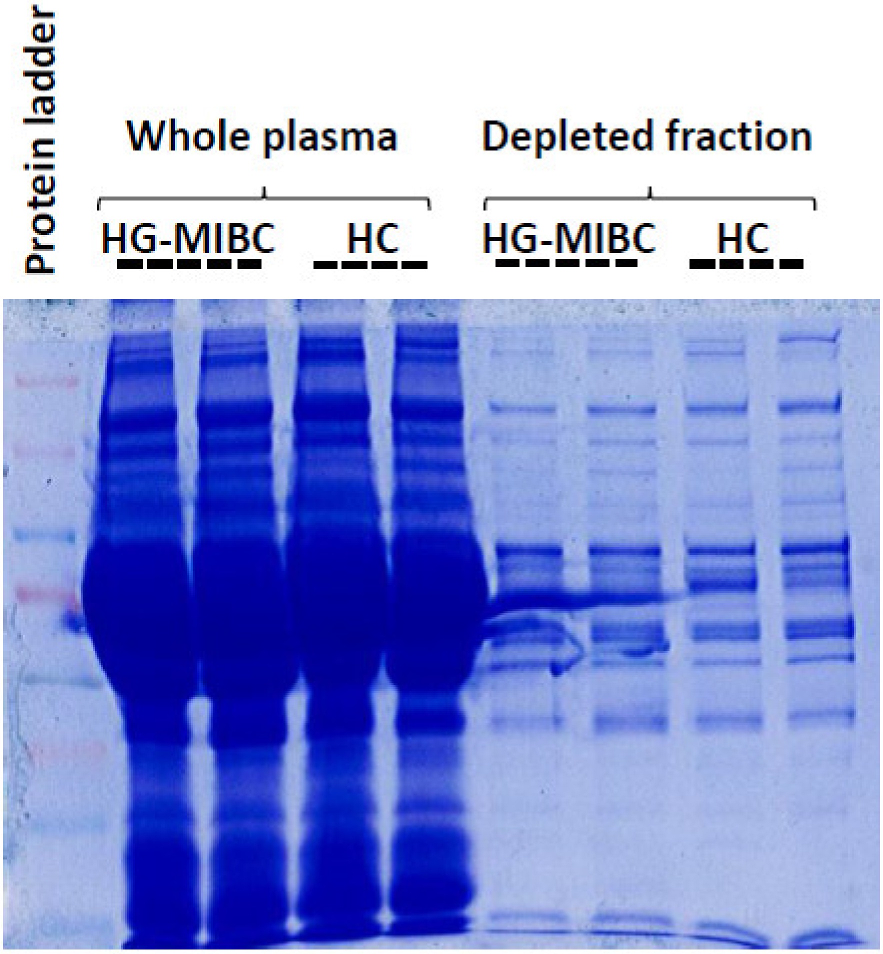

Figure S1. Depletion of plasma samples. 8% Coomassie blue-stained SDS gel showing 6 µg of whole plasma and depleted fractions from high grade muscle-invasive bladder cancer patients (HG-MIBC) and healthy controls (HC).

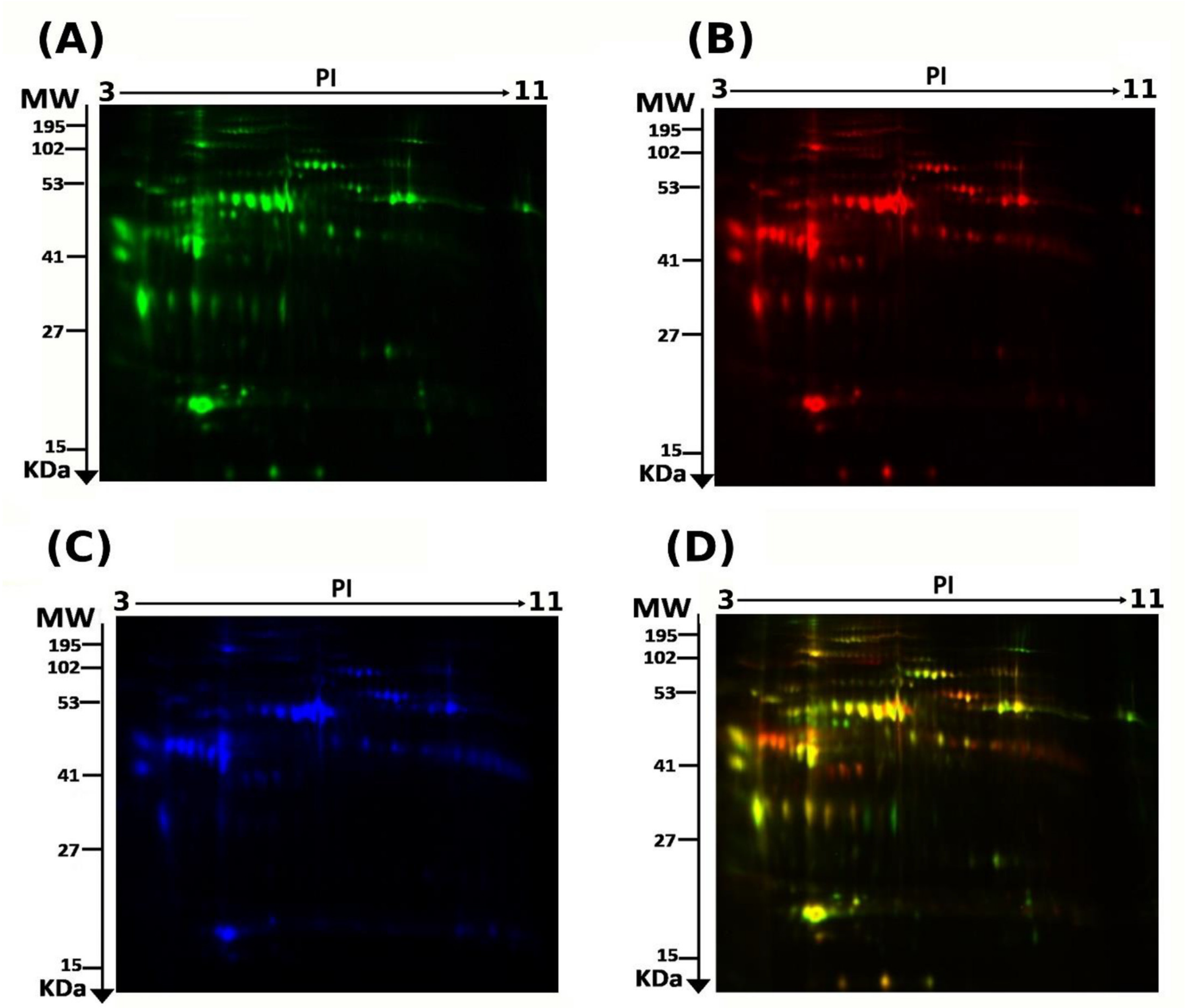

Figure S2. Protein expression profiling by 2D-DIGE: Images (**A**,**B**) for high grade tumor and healthy control labeled with Cy3, Cy5 dyes respectively. Pool samples (internal standard) was labelled with Cy2 dye (**C**). Image (**D**) is an overlay of 2D-DIGE gels.

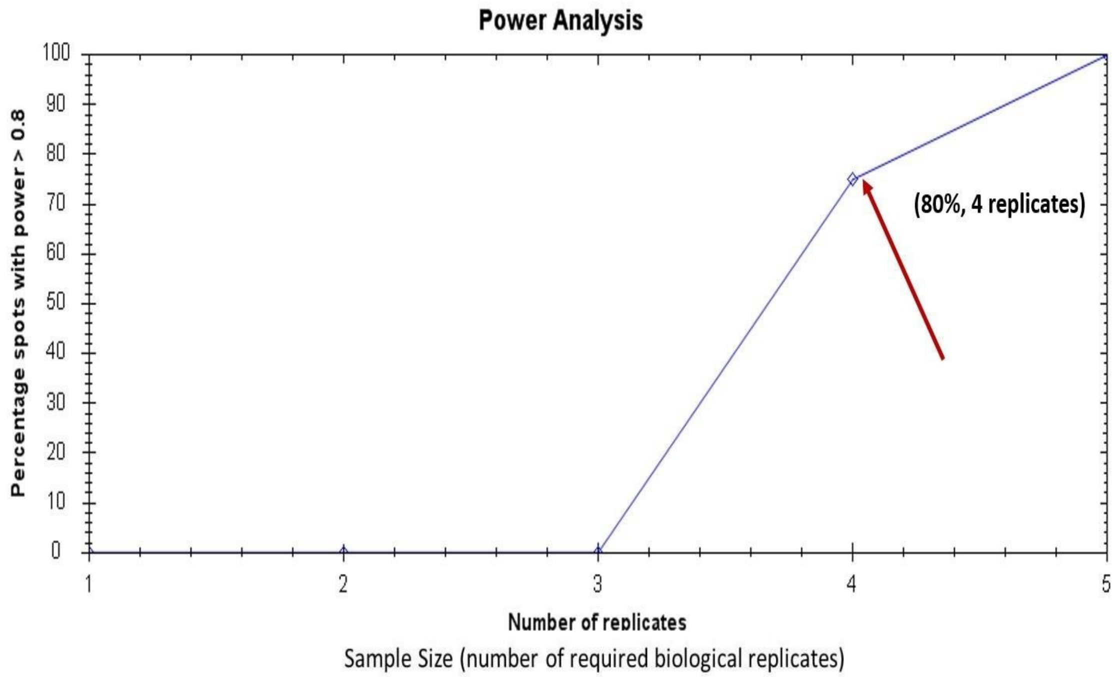

Figure S3. Determining the Sample size number. Pilot study of the power analysis using group of samples (4 replicates). A sample size of 4 was determined as sufficient to give a power of 80% by software Progenesis SameSpots Non Linear dynamics.

Table S1. Experimental design for Cyanine Dye labeling of depleted plasma proteins for DIGE analysis. discovery set, 4 high grade cancer (HG/MIBC) and 4 control samples run on four 2D-PAGE gels, Internal standards were used for normalization and created by pooling equal amount of samples.

Gels	Cyanine Dye (Cy3)	Cyanine Dye (Cy5)	Cyanine Dye (Cy2)
**1**	HG # 07	Control # 12	Pooled sample (std)
**2**	Control # 13	HG # 08	Pooled sample (std)
**3**	HG # 09	Control # 14	Pooled sample (std)
**4**	Control # 15	HG # 10	Pooled sample (std)
